# Characteristics and engagement among English-language online forums for addiction recovery available in the US

**DOI:** 10.1016/j.invent.2024.100708

**Published:** 2024-01-13

**Authors:** Jason B. Colditz, Lily H. Hsiao, Brandon G. Bergman, David W. Best, Eric G. Hulsey, Jaime E. Sidani, Bruce L. Rollman, Kevin L. Kraemer

**Affiliations:** a Division of General Internal Medicine, School of Medicine, University of Pittsburgh, 230 McKee Place #600, Pittsburgh, PA 15213, United States; b Recovery Research Institute, Massachusetts General Hospital, & Harvard Medical School, 151 Merrimac Street, 4th Fl., Boston, MA 02114, United States; c Centre for Addiction Recovery Research (CARR), Leeds Trinity University, Trinity Building, Brownberrie Road, Leeds LS18 5SD, Yorkshire, UK; d Department of Behavioral and Community Health Sciences, University of Pittsburgh School of Public Health, 130 DeSoto Street, Pittsburgh, PA 15261, United States

**Keywords:** Substance use, Alcohol use, Addiction recovery, Online, Forum, Social media

## Abstract

In developing public resources for the Networks Enhancing Addiction Recovery – Forum Activity Roadmap (NEAR-FAR), we completed a systematic observational study of English-language online forums related to recovery from alcohol or other drug addiction in late 2021. Among 207 identified forums, the majority were classified as “general addiction” or alcohol-focused, though classifications related to other substances were common on websites hosting multiple forums. Commonly used social media platforms such as Reddit, Facebook, or Quora offered easily accessible venues for individuals seeking online support related to a variety of substances. Forums were related to established recovery programs such as 12-step and SMART Recovery as well as other nonprofit and for-profit recovery programs, and to community forums without formal recovery programming. Among 148 forums with any observed user activity, the median time between unique user engagements was 27 days (inter-quartile range: 2–74). Among 98 forums with past-month posting activity, we found a median of <10 posts per week (inter-quartile range: 1–78). This study compares three metrics of observed forum activity (posts per week, responses per post, time between unique user engagements) and operationalizes forum characteristics that may potentiate opportunities for enhanced engagement and social support in addiction recovery.

## 1 Introduction

Addictive disorders related to alcohol, tobacco, and other substance use represent a serious global health burden (Rehm and Shield, 2019), are often comorbid with other mental and physical health problems (US Substance Abuse and Mental Health Services Administration, 2023), can require several attempts to resolve—specifically for those with more severe variants (Kelly et al., 2019)—and are under-treated globally (United Nations Office on Drugs and Crime, 2023). Peer support groups play a pivotal role in supporting *recovery* from addictive disorders, including among a majority of people with addiction who are unlikely to engage in clinical treatment for a number of reasons (e.g., inadequate access to care, lower severity addiction phenotypes). For example, Alcoholics Anonymous (AA) is active globally and promotes the peer-based 12-step approach, which is broadly effective for mitigating alcohol use disorder and associated problems (Kelly et al., 2020). However, as AA and other peer recovery groups were disrupted through COVID-19 social distancing and isolation, in-person activities increasingly moved into digital environments (Bergman and Kelly, 2021). Prior to the COVID-19 pandemic, 1 in 10 US adults who resolved a substance use problem had used recovery-oriented online technology (Bergman et al., 2018). Increases in the availability and use of digital technologies for peer support profoundly changed the landscape of addiction recovery. Such changes are reflected in the release of public resource guides by United States (US) organizations such as the Substance Abuse and Mental Health Services Administration (SAMHSA;, 2020) and American Society for Addiction Medicine (ASAM;, 2020), which notably included listings for virtual support groups and recovery-oriented social media forums.

Public health resources related to this topic appear encouraging, but evidence is limited for the effectiveness of *online forums* (e.g., social media groups, recovery websites with community interfaces) in reducing addiction-related morbidity or contributing to favorable recovery outcomes. While online addiction support forums have been a topic of scientific research since at least the turn of the century (Madara, 1999), there is insufficient prior research to delineate what quantity or quality of forum activity might encourage sustained engagement and contribute to favorable recovery outcomes (Bergman and Kelly, 2021). Using such forums is associated with a variety of individual factors such as family composition or being early on in a recovery process (Gilbert et al., 2022), and the social support exchanged in forums is associated with favorable outcomes such as reduced relapse rates (Liu et al., 2020). However, generalizability of research in this milieu is hindered by the heterogeneity of forums where support is exchanged. This heterogeneity is reflected in technological platform affordances (e.g., discussion interfaces, ancillary features) as well as community affordances (e.g., populations served, moderator presence), which influence the ways that forum users can safely and meaningfully engage with peers. In light of these social-technological considerations, it is timely to review the landscape of available recovery support forums that may confer potential benefits (e.g., developing coping skills, supportive networks) as well as risks (e.g., privacy concerns, passive or ineffective use of online resources) among people who are pursuing addiction recovery (Bergman and Kelly, 2021). It is also important to consider that *clinical recovery* (i.e., remission of symptoms) and *personal recovery* may be understood differently across countries and cultures (Slade et al., 2008). As this is a heterogeneous global phenomenon that has been primarily researched in a US context (Inanlou et al., 2020), it is important to consider that online recovery forums that may be beneficial in a US context may function differently across other cultures and global populations.

To advance research around English-language addiction recovery forums, we report on the development of an observational taxonomy that accounts for recovery forum characteristics and overall user engagement. A parallel use case for this taxonomy included our development of an interactive dashboard (Networks Enhancing Addiction Recovery - Forum Activity Roadmap; NEAR-FAR), which allows individuals to identify active forums that match their perceived needs and interests. Our focus on recovery forums complements established public resources in the US, which have broader health services foci (e.g., SAMHSA, ASAM) as well as recent work that resulted in a public dashboard for smartphone applications related to addiction (Lagan et al., 2021). The taxonomy from the present study can serve as a basic reporting framework in future observational studies of recovery forums. The identified engagement metrics may also be important to control for in future studies of recovery forum effectiveness. Thus, the present work presents a taxonomy of recovery forum characteristics and engagement metrics. This taxonomy is used to systematically describe the observed landscape of online addiction recovery forums that are relevant to English-speaking populations, primarily in the US.

## 2 Material and methods

We report on a cross-sectional, observational study of online recovery forums, which we conducted whlile developing a taxonomy that was integrated into the NEAR-FAR dashboard (https://onlinerecovery.pitt.edu/near-far). Through this process, we developed a pragmatic approach to systematically search for, catalogue, and review online recovery forums in the English language. Where possible, we ascertained engagement metrics (e.g., posts per day), other forum features (e.g., associated chat rooms, linkage to in-person meetings), and specific demographic groups that forums intended to support (e.g., gender identity, secular or specific religion).

### 2.1 Data collection

Beginning in November 2020, we used a systematic process to document English-language websites that provided support for substance use recovery (including alcohol, nicotine, and other drugs). For the purposes of this work, we operationalized *recovery* as “a process related to stopping or reducing use of an addictive substance while making other changes to improve overall health and wellbeing.” Specifying “stopping or reducing use” in this definition narrowed the scope so as not to include online resources that actively support or encourage substance use (e.g., offering advice on obtaining, preparing, or using particular substances).

We first reviewed extant online support resource guides from US-based organizations including SAMHSA, ASAM, and the National Institute on Drug Abuse (NIDA). We identified any resources categorized as peer support sites. This led to a pool of 48 unique websites. For each identified site, we navigated to related pages (e.g., resources, frequently asked questions, help) that linked to other recovery sites, which were added to our listing. We continued this process recursively for newly identified sites, which resulted in 74 additional websites related to addiction recovery support. We then conducted Google searches for additional websites using search strings related to addictive substances and recovery, and online support modalities. We developed search strings that specified “alcohol” or “alcoholic” as anchor terms from a common US lexicon, while using terms of “addiction” and “substance use” to broaden the search to other substances. We avoided “drug” so as to reduce irrelevant results related to prescribed pharmaceuticals and also did not include specific drug terminology to maintain feasibility of search procedures. Terms also reflected common US terminology including “substance use” as a low threshold and “substance abuse” indicating a higher severity of use, as is reflected in the SAMHSA moniker (i.e., *Substance Abuse* and Mental Health Services Administration). We also incorporated various terms related to online forums (e.g., discussion board, chat, community). After pilot testing and discussing various strategies as a group, we finally synthesized eight distinct search strings using various combinations of these terms (Table 1). We reviewed listings for the first three pages of results for each search (i.e., 30 most relevant listings per search). Due to substantial overlap among sites identified via search results and recursive identification procedures, it was not feasible to enumerate the exact number of sites that were excluded as duplicates across search and retrieval procedures. Google-based searches elicited 127 additional websites, bringing the total to 249 websites considered for abstraction procedures. While we were primarily interested in identifying forums, we also identified whether sites linked to in-person meetings, online meetings, clinical/treatment services, synchronous chat rooms, blogs, or email listservs.

**Table 1:** Search parameters.

**Google search queries**
Alcohol online help group
Substance abuse online forum
Addiction online support group
Substance use discussion board
Online recovery support forum
Alcoholic chat
Recovery chat
Online addiction community

**Website search keywords**

Addiction
Alcohol
Drug[s]
Recovery
Sobriety
Substance [ab]use

### 2.2 Inclusion and exclusion

As new sites were identified, they were screened for inclusion and exclusion into abstraction procedures. Sites were included if they had at least one *online forum*, which we defined as “an interactive website feature allowing three or more people to correspond via asynchronous text dialog that remains on the website after leaving.” This stands in contrast to a chat room, where synchronous text is accessible only during a single real-time chat but not after the room closes. Forums could be hosted directly on a website or on an external social platform (e.g., Facebook). Sites were excluded if (1) all site content was unavailable or unreadable (e.g., expired web domain, not in English), (2) resources were not directly related to recovery from substance use (e.g., gambling, Internet addiction, substance-related without a recovery focus), (3) resources were directed toward people affected by others' substance use (i.e., concerned family and friends), or (4) the presence of a forum could not be confirmed due to the website requiring user authentication or other barriers to accessing relevant content. This fourth exclusion criterion was necessary to maintain ethical compliance for analysis of publicly available data and resulted in excluding at least two sites with known forums (InTheRooms and Discord). We accessed sites using the Chrome web browser set to “incognito mode” to ensure results were not influenced by extraneous factors (e.g., past search history, being logged into social media platforms).

Within websites that contained multiple forums for various other health topics, we searched for relevant forums via the site search box (where available) using the keywords identified in Table 1. If a search box was not available, we navigated through available listings (e.g., front page, table of contents) to identify forums related to substance use or addiction. Each forum was then catalogued in a spreadsheet and data were abstracted according to our coding procedures.

### 2.3 Codebook development and data abstraction

We were unable to find comprehensive coding frameworks for online recovery forums in the extant literature. With conceptual feedback from all authors, we developed broad coding categories related to target population, privacy and accessibility, forum characteristics and features, and forum engagement. Over two months, two coders (LH and JC) reviewed approximately 10 new forums per week for purposes of iterative codebook development. With each iteration, definitions were clarified and emergent categories were added until coders agreed that thematic saturation was sufficient to finalize the codebook. Previously coded forums were re-reviewed using the final coding structure, as follows:

• ***Specific substance focus*** indicated the presence of descriptions or labels indicating that the forum focused on a particular substance (i.e., nicotine, alcohol, cannabis, hallucinogens, opioids, stimulants, other). Codes differentiated a *primary* focus in cases where the site indicated a specific substance or user type (e.g., “a site for alcoholics”) and *secondary* foci if more than one specific substance was identified (e.g., “people recovering from alcohol, opioids…”). Forums that identified no specific substances or that focused on other general substances (e.g., “…and other substances”) were respectively coded as *primary non-specific* and *secondary non-specific*.
• ***Population*** was an open-ended field to collect notes about forums that were explicitly directed toward specific populations. Over the course of coding, this inductively resulted in categories of *gender*, *minoritized populations*, *spiritual/religious orientations*, and *professions*.
• ***Visibility*** indicated the quality of public or private access to forum content. Forums were considered *private access* if no user content was publicly visible on the forum (e.g., content grayed out, blocked by a signup/login page), *public access* if all forum content was publicly visible, and *semi-public* if some but not all content was visible (e.g., some forum views or topics were designated as “members only”). We also assessed whether the website charged money for *subscription access* to forum content.
• ***Engageability*** indicated the extent to which a public user (i.e., not logged in to the site) could actively engage with forum content. *Public engagement* was indicated where site visitors could post or comment without logging in. *Authenticated engagement* was indicated where only logged-in users could post or comment.
• ***Content threading*** indicated the structure of forum content as either *threaded*, where responses were organized hierarchically under other responses, or *flat*, where responses to posts could not be directly responded to and/or responses were organized into a basic chronological timeline.
• ***Content feedback*** indicated the ability to provide *basic positive feedback* (i.e., liking, favoriting, upvoting), *basic negative feedback* (i.e., disliking, downvoting) and *complex feedback* (i.e., emoji faces, symbols) toward posted content.
• ***User profile*** indicated the extent of information available via user profiles (if present). In addition to including a username, *basic* profiles included a visual avatar or short biographical description. *Detailed* profiles also included any of: gender, date of birth, geographic location, contact information (e.g., phone number, email address), occupation, or professional qualifications.
• ***User labels*** indicated the availability of “badges” or “flair” attached to user profiles, which indicated a user's social standing within the forum. Types of labels included *authority* (e.g., forum moderator, top-rated commenter), *seniority* (i.e., duration of activity or service in the forum), and *sobriety*. The sobriety code was used to identify forums that had such tracking functionality available and visible to other users, which could either be simple date labels (e.g., “sober since…”) or a rolling counter to quantify the passage of time (e.g., “…days sober”).
• ***Networking*** indicated features for connecting with groups of users directly or through shared interests. *Peer networks* were identified as forums having “follow” or “friend” features that allowed users to curate lists of other users for easier access to their content. Some friend networks were *public*, meaning that these lists were visible to people outside the network. Other networking features included *@’ing* (i.e., including another user's handle to include them in dialog), *hashtagging* (i.e., using “#hashtag” notation to categorize and increase discoverability of content), and *categorical tagging* (i.e., attaching labels to content based on categorically limited options).
• ***Content moderation*** indicated that the forum included a listing of content moderators or a description of active moderation practices, or that we could identify examples of moderation activity in the forum (e.g., a user with a moderator label responded to another user).

After the codebook was finalized, one coder (LH) reviewed each forum and all forums were re-reviewed and codes verified in meetings with the lead investigator (JC). Data collection and abstraction took place between November 2020 and December 2021 and adjudication took place in parallel via weekly teleconference meetings. Frequencies and distributions of codes were analyzed to present descriptive statistics. Coders also recorded memos throughout abstraction, noting emergent themes that were apparent across forums and idiosyncrasies that were unique among forums. These notes are synthesized as qualitative descriptions in the results section.

### 2.4 Assessing user engagement

For forums that were publicly accessible or that publicly reported usage data, we assessed the rate of user engagement in three ways. Due to divergent forum configurations and limitations (e.g., limited post history, missing timestamps, inability to sort chronologically), a single metric did not work across all forums. Assessments of forum engagement were completed between 7/7/2021 and 11/7/2021. Primary engagement metrics focused on:

• **Posts-per-week (PPW)** was ascertained by counting the total number of forum posts made in the past seven calendar days. If there were no engagements in the past week due to low activity, we reviewed the past month of activity and divided the total by four to estimate a fractional number of weekly engagements. This was coded as 0 = *inactive* if there were no posts in the past month.
• **Responses-per-post (RPP)** was calculated for forums that had a nonzero PPW value. The number of responses was summed across all posts that were made in the past week (or past month if no posts in the past week) and then divided by the number of posts to calculate an average RPP.
• **Time between unique user engagements (TBU)** included posts and/or responses as engagements, depending on the forum structure. Engagements were reviewed reverse-chronologically (i.e., newest to oldest) and unique users (i.e., screen names) were tabulated in a notebook until 10 were identified. We calculated the time, down to quarter-hour granularity, between the most recent user engagement and the tenth most recent user engagement (divided by 10). If there were fewer than 10 forum users in the past year, this metric was not calculated.

In cases where multiple metrics were available, we calculated pairwise correlation coefficients to compare rankings of forum engagement across available user engagement metrics. As engagement data were not assumed to be normally distributed, we report descriptive statistics as non-parametric measures of central tendency (i.e., Median, Inter-Quartile Range [IQR]) and Spearman rank correlations, as calculated in R statistical software.

## 3 Results

As of November 2021, we abstracted data from 249 English-language websites related to alcohol, tobacco, or other drug addiction recovery support. These sites provided linkage to one or more of: in-person meetings (*n* = 49), online meetings (*n* = 87), clinical/treatment referrals (*n* = 83), synchronous chat rooms (*n* = 38), blogs (*n* = 76), and email listservs (*n* = 10). Twenty-five sites provided linkage to forums but did not host forums on the website nor on social media platforms. Recovery forums were identified for 66 sites. Of these, 49 sites had only one associated forum and 17 had more than one (Table 2). We identified 207 distinct forums in total.

**Table 2:** Websites with multiple alcohol, tobacco, or other drug addiction recovery forums.

Site name	Domain	Forums identified
Reddit	reddit.com	37
Facebook	facebook.com	29
Support groups	supportgroups.com	19
Quora	quora.com	17
Addiction recovery guide	addictionrecoveryguide.org	13
Talk recovery	talk.recovery.org	11
Drug abuse	drugabuse.com	9
Drugs forum	drugs-forum.com	9
Daily strength	dailystrength.org	7
MedHelp	medhelp.org	4
PsychForums	psychforums.com	4
7 cups of tea	7cups.com	3
Inspire	inspire.com	3
Harm reduction for alcohol	hams.cc	2
Miracles in progress	12stepforums.net	2
Moderation management	moderation.org	2
Patient Info	patient.info	2

Of the 49 sites with a single associated forum, 15 used Facebook to host their forums off-site, which is included in Facebook's total count in Table 2. The other 14 Facebook forums were those with no external organization identified. While there were no official organization forums on Reddit, there were dedicated subreddit communities for members of AA as well as “Atheist Twelve Steppers” and SMART Recovery programs. Sites with 9 or more forums generally included non-specific addiction recovery forums, alcohol-specific forums, and multiple forums for other classes of substances.

### 3.1 Substance and population focus

Among the 207 identified forums, 98 (47.3 %) had primary focus on a specific category of substance, with alcohol the most common primary focus, and the remainder were non-specific or focused on multiple substances (Table 3). Among these forums, 153 (73.9 %) were publicly visible, 13 (6.3 %) were semi-public (i.e., some but not all content was visible), and 41 (19.8 %) were private. Eight private forums required paid subscriptions to access content and one other subscription-based site allowed access via a free trial membership. Two paid subscription websites used the Facebook platform to host forums. Among the six subscription sites where the primary focus was alcohol use, three were for women and one of those was specifically for mothers. The remainder of subscription-based forums were non-specific (i.e., focused generally on addiction recovery) and were not directed at a specific demographic population.

**Table 3:** Substance focus of 207 identified forums.

Substance	Primary focus n (%)	Any focus n (%)
Alcohol	44 (21.3)	67 (32.4)
Opioids	14 (6.8)	28 (13.5)
Stimulants	17 (8.2)	21 (10.1)
Nicotine	12 (5.8)	18 (8.7)
Cannabis	10 (4.8)	15 (7.2)
Hallucinogen	1 (0.5)	2 (1.0)
Non-specific	100 (48.3)	121 (58.5)

Note: *Primary focus* sums to 95.7 % because 9 forums had only secondary foci but were not broad enough to be considered primarily non-specific. *Any focus* includes primary and secondary foci and sums to 131.4 % because multiple secondary foci could be identified per forum.

With respect to the intended populations of forums, we further categorized observational notes as related to gender, minoritized populations, spiritual/religious orientation, and profession. We did not identify any forums directed toward specific age groups. While forums were generally inclusive of women, nine forums were directed specifically toward women. Notably, seven of these were private and three of these required a paid subscription to access. There was also one private forum for sexual or gender minorities (“LGBTQ+ Forum” on SMART Recovery website) and one for racial minorities (“Sober Black Girl Squad” website forum). While we were unable to clearly differentiate between religious, spiritual, or agnostic/atheistic approaches to recovery, we could identify seven forums explicitly oriented toward Buddhists (n = 3), Christians (2), Jews (1), or Atheists (1); six additional forums self-described as “spiritual but not religious.” At least four secular recovery programs were identified as having online forums (SMART Recovery, SoberRecovery, LifeRing, SOS Recovery Community). With respect to professions, we identified one forum each for pilots and airline workers, military veterans and first responders, and medical and mental health professionals. We identified numerous forums unofficially affiliated with 12-step programs (Alcoholics Anonymous, Narcotics Anonymous, Dual Diagnosis Anonymous, Nicotine Anonymous), as well as forums for SMART Recovery (available via website, Reddit, and Facebook), HAMS: Harm Reduction for Alcohol (website and Facebook groups), Women for Sobriety (website), and Moderation Management (website). While these were free of cost, a number of subscription-based websites with forums were also prominent in our Google search results (e.g., Tempest, Monument, Soberistas).

Among the 166 forums that were not private (Table 4), 30.7 % were hosted either as “subreddits” on the Reddit platform (*n* = 36, 21.7 %) or as “spaces” on Quora (*n* = 15, 9.0 %). These platforms were similar in that they require user authentication to engage, have threaded content, and allow positive and negative feedback. While similar in features, Quora differs in that it is designed specifically as a question-and-answer site and publicly lists both following and follower networks, whereas Reddit users do not know who other users are following. Reddit profiles are also more basic, lacking the names, identifying photos, and detailed background information observed on Quora profiles.

**Table 4:** Characteristics and features of non-private forums (*n* = 166).

Characteristics and features	Frequency	%
**Engageability**		
Public (i.e., no login required)	15	9.0
Authenticated	151	91.0
**Discussion threading**		
Flat (i.e., not threaded)	83	50.0
Threaded	80	48.2
Functionality broken or unclear	3	1.8
**Content feedback**		
Positive only (e.g., thumbs up)	45	27.1
Positive and negative	52	31.3
Complex (e.g., emoji)	8	4.8
Not identified	61	36.8
**User seniority labels**		
Authority (e.g., moderator)	37	22.3
Duration of membership	34	20.5
Both authority and duration	66	39.7
Not identified	29	17.5
**User sobriety labels**		
Date of sobriety	6	3.6
Duration of sobriety	5	3.0
Not identified	155	93.4
**User profiles**		
Basic	63	38.0
Detailed	62	37.4
Not identified	41	24.7
**Following/follower networks**		
Privately listed	37	22.3
Publicly listed	47	28.3
Not identified	82	49.4
**Content tagging**		
Categorical labels	47	28.3
Hashtags (i.e., #*hashtag*)	4	2.4
Not identified	115	69.3
**User mentions (i.e., @*username*)**		
Identified	62	37.3
Not identified	104	62.7
**Moderation**		
Identified	97	58.4
Not identified	69	41.6

### 3.2 Engagement metrics

We report on forum engagement using three available metrics of PPW, RPP, and TBU. Among the 207 identified forums, PPW was ascertainable for 98 forums (47.3 %), which included 27 private Facebook groups that publicly reported posts-per-month metrics. PPW was not ascertainable for 15 forums due to privacy and was coded as “inactive” in 94 cases where there were no detectable posts in the past month. RPP was ascertained for 146 forums (70.5 %; 88.0 % among non-private forums) and TBU was ascertained for 148 forums (71.5 %; 89.2 % among non-private forums). In forums where PPW, RPP, or TBU were able to be ascertained for pairwise comparison, we calculated descriptive statistics and intercorrelations for engagement metrics (Table 5).

**Table 5:** Descriptive statistics and Spearman correlations among engagement metrics.

				Spearman *r*	
	n	Median	IQR	RPP	TBU
Posts-per-week (PPW)	98	9.63	1.25–78.25	0.46 (n = 61)	−0.91 (n = 66)
Responses-per-post (RPP)	61	4.00	1.38–9.84	–	−0.50 (n = 56)
Time between unique user engagements (TBU; in days)	148	26.94	2.30–74.02	–	–

Abbreviations: IQR, interquartile range.

Among 98 active forums with PPW data, the most active forum (Reddit's r/StopDrinking) comprised 24.7 % (*n* = 1992) of all posts included in PPW calculations observed across forums (*N* = 8060). The top five most active forums comprised 47.0 % of total posts observed. These included: r/Leaves (637 PPW), Facebook's “Narcotics Anonymous Recovery Group” (530 PPW), r/QuittingKratom (324 PPW), and r/StopSmoking (308 PPW) forums.

Among 61 active forums with RPP data, the most responsive forum (My Way Out Forums) had 27.5 responses per post, though there were few posts overall (0.5 PPW). The top five also included Sober Recovery (20.7 RPP), r/Methadone (20.2 RPP), NetMums Forum (14.3 RPP), and r/Suboxone (13.7 RPP). Notably, of these five, the three non-Reddit forums had PPW < 1, as responses were centralized around fewer and older posts. Nine forums (14.8 %) had an RPP value of less than one, with the lowest ratio of four posts per one response. Where both metrics were available, we found a small positive correlation between PPW and RPP (Table 5).

As compared to PPW or RPP metrics that had a floor effect at one post per month, TBU were ascertainable for a larger number of lesser-active forums. The maximum observed TBU value was 323 days and the median TBU value indicated nearly a month between unique user engagements (Table 5). However, TBU could be better expressed in minutes for the most active forums. The five forums with the lowest TBU at the time of review were: r/StopDrinking (1.5 min), Talking Sober (3.0 min), r/Leaves (18.0 min), r/Methadone (25.5 min), and the UK National Health Service's “Quit Smoking Support Group” on Facebook (28.5 min). Overall, TBU had a strong inverse correlation with PPW and a modest inverse correlation with RPP (Table 5).

## 4 Discussion

We developed the publicly available Networks Enhancing Addiction Recovery - Forum Activity Roadmap (NEAR-FAR) website and resource guide, which provides a dashboard of English-language forums identified through the present study. Using the PPW metric, we categorized forums by engagement level (simplified into groups as *low* [PPW ≤ 7], *moderate* [7 < PPW < 70], and *high* [PPW ≥ 70]) so that more active sites appeared higher in the listing and sites with no past-month posts were considered inactive. NEAR-FAR also allows for forums to be filtered by specific substance, access (i.e., public, private, subscription), associated features (e.g., in-person meetings, chatrooms, mobile apps), and population focus (e.g., spiritual, religious, gender, LGBT+). This emerging taxonomy represents advancements in systemization and transparency as compared to extant public health guides, though further improvements are warranted (e.g., incremental updates to account for changes in forums over time, engagement metrics that account for distinct types of social support). Scientifically, this work further contributes to our understanding of common characteristics and observable engagement levels for online addiction recovery forums. These characteristics will be important to consider in future studies that assess the effectiveness of online support in enhancing addiction recovery outcomes.

Engagement metrics provide an important perspective into the availability or timeliness of peer support on particular forums. Even after excluding forums that were broken or inaccessible, a substantial number of forums demonstrated what we would consider relatively low levels of engagement. While there was a wide range of posting activity, a value closer to the upper quartile of PPW (e.g., > 70 PPW or about 10 posts per day) could be a reasonable benchmark to consider a forum as having regular engagement. The TBU metric allowed for a more nuanced understanding of groups with lower levels of engagement. However, we would expect this metric to be highly variable in forums with a large number of active users, such that it may be affected by weekly or daily forum usage patterns. Reassuringly, TBU was strongly correlated with PPW and so either metric might be used to estimate engagement, depending on the structure and activity level of the forum. While RPP remained conceptually valuable to complement PPW, this metric was ascertainable in fewer than 30 % of non-private forums due to lack of discussion threading or forum navigation features. RPP was also exceptionally high in some forums with relatively older and fewer posts, particularly on sites where posts were organized into topical categories. Thus, RPP or similar metrics related to responsiveness should be contextualized with a complementary forum engagement metric. Future work should test whether users of highly engaged forums exhibit relatively better recovery outcomes compared to users of less-engaged forums.

A number of websites and forums were directed toward special populations in recovery. It was notable that forums for women required a paid subscription relatively frequently. Online forums primarily for women facilitate easy access to a potentially valuable social support in recovery (Chambers et al., 2017) and women-led entrepreneurial ventures can be characteristically benevolent while innovating social change through novel technologies (Borquist and de Bruin, 2019). While it was beyond the scope of our investigation to detail the founding principles of each forum, the presence of women entrepreneurs in the online recovery milieu was salient. While Gilbert et al. (2022) identified separate predictors for men and women regarding their relative engagement in online recovery forums, additional research is needed to understand the costs and potential benefits/risks that are unique to participating in gendered and other identity-exclusive online forums for addiction recovery.

Several recovery-oriented websites and organizations used Facebook groups to host both publicly visible as well as private forums. Access to these groups was in some cases limited to paying subscribers of external websites. As Facebook is among the most frequently used social platforms in the US (PEW Internet Research Center, 2021), this platform might serve as a relatively easy to navigate on-ramp to online recovery support. Yet recovery forums we identified on Facebook had relatively lower levels of engagement than those on Reddit, which has a smaller overall user base. This disparity may relate to differences in platform privacy and the higher degree of user anonymity offered in Reddit (Nagel and Frith, 2015), which could be appealing given the particularly sensitive nature of addiction recovery. Reddit appears to be a popular choice for individuals seeking online addiction recovery support as it offers the greatest number of publicly visible forums. Visibility of forum content is a particularly important consideration, given that support seekers often passively observe (colloquially “lurk”) on forums for a period of time prior to engaging (Chambers et al., 2017). This situates Reddit forums as persistent sources of informational support, similar to Quora's question-and-answer forums, but with a greater degree of user anonymity and opportunities to provide a broader range of support (e.g., emotional support, personal narratives). While engagement metrics do not account for specific types of social support that may be helpful in recovery, further research should focus on how different platforms or forums are amenable to different types of support (e.g., informational support on Quora, emotional support on Reddit). A study by Colditz et al. (2023) confirmed the presence and overlap of distinct subtypes of social support on the r/StopDrinking forum, and quantifying these may provide additionally useful metrics of engagement.

Two Reddit forums (r/StopDrinking and r/StopSmoking), among 11 forums in total, provided sobriety tracking features. While the Reddit “sober badges” had been included in prior observational work (Tamersoy et al., 2017), there remains a research gap related to the impact of such features. While sobriety trackers are potentially valuable tools in recovery (Hoeppner et al., 2017), having them publicly displayed could also lead to social hierarchies around success or failure to maintain abstinence. This is an important consideration for forums, as some users may find this motivating while others (particularly individuals aligned with harm reduction approaches or having trouble maintaining abstinence) may find it discouraging. More robust behavioral tracking features are available via numerous mobile applications (Hoeppner et al., 2017). Thus, sobriety trackers may function primarily as a reinforcer of social norms (i.e., abstinence) and social hierarchies, and more research is warranted on the potential benefits and drawbacks of integrating them into recovery forums. Similarly, the ability to “upvote” and “downvote” content can function as a way for online communities to reinforce norms of etiquette and forum behavior. These features were common and were integral to forum functionality (e.g., higher-scoring posts natively appeared first in Reddit forum feeds). In forums with unclear or inconsistent moderation practices, these features may be particularly important in promoting content that aligns with a naturally emerging community ethos and demoting content that is hostile or potentially harmful to other community members (Nagel and Frith, 2015).

A limitation of this work was its cross-sectional approach. While this limited the timeliness of findings, this approach maintained the feasibility of studying a broad swath of forums and evaluating multiple engagement metrics. We hope that future observational studies of online support forums will report on community-level metrics like PPW, RPP, and TBU, where possible, to contextualize important social dynamics. For example, Colditz et al. (2021) found that engagement on the r/StopDrinking forum exhibited predictable peaks of activity around “Dry January” and that engagement was significantly reduced early in the COVID-19 pandemic. While we avoided January engagement data in the present study, there may be other temporal considerations related to months (e.g., National Recovery Month in September, Sober October) or smaller temporal patterns such as weekday versus weekend use that might influence forum engagement and responsiveness. Events like Dry January can also serve as on-ramps to engaging in online peer support, presenting opportunities for community engagement (Russell et al., 2022). Additional research is needed to determine whether these community initiatives result in longer-term engagement in online peer support and sustained reductions in alcohol or substance use. Another limitation is that we could not assess private forums in detail, so our results may not reflect the full range of forum characteristics or engagement. We are also aware of at least two websites (InTheRooms and Discord) that host recovery forums, but these were not included in analysis as no specific forums could be identified from the public websites. Our results may also be affected by the selection of search terms used for data collection, given that we used “recovery” as an organizing framework for NEAR-FAR – rather substance use related health behavior change more broadly (e.g., harm reduction) - and also included alcohol as one specific substance while terms for other specific substances were not included. As a result, we may have missed some forums embedded in sites that did not align with this recovery concept or that were related to specific substances other than alcohol. Thus, these results may not generalize to non-English speaking forums or those focusing on populations outside the US. As non-recovery sites were excluded according to our criteria, future research might use a broader definition to incorporate sites that are aligned with other harm reduction approaches. Subsequent to our analyses, we were made aware of two additional forums (r/QuitVaping on Reddit and “Health and Recovery” on Bluelight), which were not identified through our search strategies. These and other newly identified forums will be added to the NEAR-FAR dashboard as they are identified. Thus, while the initial dashboard represents a substantial improvement over contemporary guides to addiction recovery forums, this initiative will benefit from additional support of community partners and researchers to maintain a more comprehensive and timely listing of recovery forums.

Finally, while this work provided a foundational understanding of online recovery forum characteristics and engagement, we cannot place clinical value on these features. Whether engagement in recovery forums results in improved alcohol or substance use outcomes remains unclear (Bergman and Kelly, 2021), as do the possible effects of particular forum features or higher levels of engagement. Given the number of forums dedicated to various nonprofit and for-profit recovery programs, there is a strong impetus to generate evidence for their effectiveness in promoting favorable recovery outcomes and, of equal importance, to identify any null or iatrogenic effects of online forum use. In the interim, we also encourage digital health researchers to report on operational characteristics and engagement metrics of forums that are being studied. This body of work should ultimately inform strategies to develop and maintain widely accessible forums as on-ramps to addiction recovery.

## 5 Conclusions

Social media platforms that are commonly used in the US, such as Reddit, Facebook, or Quora may offer an easily accessible venue for individuals seeking online support related to a variety of addictive substances. We identified forums related to 12-step and SMART Recovery programs, other nonprofit and for-profit recovery programs, and social forums unaffiliated with recovery programs. Among 207 distinct forums identified, the majority were classified as “general addiction” or alcohol-focused, though forums related to other substances were common on websites hosting multiple forums. Among 148 forums with observable user activity, nearly a month transpired between unique user engagements. Among 98 identified forums with any past-month activity, we found a median average of fewer than 10 posts per week, indicating many “active” forums with reasonably infrequent activity. We suggest that future studies in this milieu consistently report descriptive engagement metrics (e.g., posts per week, responses per post) to quantify potentially supportive peer engagement. Such metrics will be important for comparing across studies and for identifying the effects of online forum use on health outcomes.

## Funding

This work was supported by a grant from the Staunton Farm Foundation (2020–2022; Pittsburgh, PA). Dr. Colditz was supported by a 10.13039/100006108National Center for Advancing Translational Sciences training grant (TL1 TR-001858).

## Declaration of competing interest

The authors declare that they have no known competing financial interests or personal relationships that could have appeared to influence the work reported in this paper.
